# The Mediating Role of A_2A_ Adenosine Receptors in the Mitochondrial Pathway of Apoptotic Hippocampal Cell Death, Following the Administration of MDMA in Rat

**DOI:** 10.18869/nirp.bcn.8.4.317

**Published:** 2017

**Authors:** Masoomeh Bakhshayesh, Fereshteh Golab, Fatemeh Kermanian, Mehdi Mehdizadeh, Amir Reza Katebi, Mansooreh Soleimani, Farzaneh Mohammadzadeh, Ronak Shabani, Elham Movahed, Majid Katebi

**Affiliations:** 1. Cellular and Molecular Research Center, Iran University of Medical Sciences, Tehran, Iran.; 2. Department of Anatomy, School of Medicine, Alborz University of Medical Sciences, Karaj, Iran.; 3. Department of Anatomy, School of Medicine, Iran University of Medical Sciences, Tehran, Iran.; 4. Department of Educational Psychology, Faculty of Psychology & Educational Sciences, Allameh Tabataba’i University, Tehran, Iran.; 5. Department of Anatomy, School of Medicine, Hormozgan University of Medical Sciences, Bandar Abbas, Iran.

**Keywords:** Ecstasy or MDMA, Neurotoxicity, Adenosine receptor, Agonist of A_2A_ receptor, Antagonist of A_2A_ receptor

## Abstract

**Introduction:**

The 3,4-methylenedioxymethamphetamine (MDMA, ecstasy) is a popular recreational drug and a major source of substance abuse, which ultimately leads to sensations of well-being, elation and euphoria, moderate derealization/depersonalization, and cognitive disruptions, as well as intense sensory awareness. The mechanisms involved in memory impairment induced by MDMA are not completely understood.

**Methods:**

The current study used 40 Sprague-Dawley rats, weighted 200 to 250 g. Experiments were performed in four groups, each containing 10 rats. The first group of rats was used as the control, treated with dimethyl sulfoxide (DMSO). The second group was treated with MDMA. The third group was treated with MDMA and CGS (the adenosine A_2A_ receptor agonist, 2-[p-(2-carboxyethyl) phenethylamino]-5′-N-ethylcarboxamidoadenosine) (CGS 21680) and the fourth group was treated with MDMA and SCH (the A_2A_ receptor antagonist [7-(2-phenylethyl)-5-amino-2-(2-furyl-) pyrazolo-[4, 3-e]-1, 2, 4 triazolo [1,5-] pyrimidine]) (SCH 58261). The drugs in all groups were administrated intraperitoneally (i.p.) once a day for 7 days. In 5 rats of each group, following perfusion, samples were taken from hippocampi to investigate apoptosis. Accordingly, the samples were stained using the terminal deoxynucleotidyl transferase-mediated dUTP nick end labeling (TUNEL) assay kit, and studied by light microscopy. In other rats, fresh tissue was also removed to study the expression of bax and bcl-2 by Western blotting technique.

**Results:**

It was observed that the coadministration of MDMA with CGS reduced bax expression and prevented apoptosis of hippocampal cells. The coadministration of MDMA and SCH increased bax expression, and also increased the frequency of hippocampal cell apoptosis.

**Conclusion:**

The results of the current study showed that administration of CGS with MDMA decreased the common side effects associated with MDMA.

## 1. Introduction

Ecstasy (3,4-methylenedioxymethamphetamine; MDMA) is a psychoactive hallucinogenic compound considered as a source of substance abuse worldwide. It is a derivative of amphetamine related to the hallucinogenic compound, and the United Nations estimated that worldwide use of ecstasy affects 9 million people aged 15 to 64 years. Currently, there are more than 3 million ecstasy users in Europe, which represent 36% of ecstasy users worldwide (Capela, et al., 2009). MDMA produces feeling of well-being, comfort, and elation. The use of this drug also leads to a sense of moderate derealization/depersonalization, and cognitive distortions, as well as intense sensory awareness (Liechti, 2000).

Several studies reported that MDMA has the capacity to induce toxicity in neurons. Despite researches in this context, precise mechanisms of MDMA are not obvious. MDMA induces neuronal damage in several brain areas such as the hippocampus, striatum, and cortex (Battaglia, Yeh, & DeSouza, 1988; Riezzo, et al., 2010). Several factors contributed to MDMA-induced neurotoxicity, specifically hyperthermia, monoamine oxidase metabolism of dopamine and serotonin, mitochondrial dysfunction, dopamine oxidation, serotonin transporter action, formation of peroxinitrite, glutamate excitotoxicity, and importantly deficits in serotonergic biochemical markers (Broening, Bowyer, & Slikker, 1995; Fumagalli, et al., 1999; Pu, Broening, & Vorhees, 1996; Deng & Cadet, 1999; Sheng, Cerruti, Ali, & Cadet, 1996).

The hippocampus is a brain area particularly susceptible to the neurotoxic effects of MDMA. The hippocampus is critical for learning and memory (Kesner, 2007). The most complaint of MDMA is impairment in short-term memory. MDMA users show significant deficiency in delayed memory tasks, which directly associate with the increase in 5-HT receptor binding ratios. Significantly, those who take MDMA have longer reaction time to visual and auditory stimuli, lower visual recall, and lower working memory scores (Green, Mechan, Elliott, O’Shea, & Colado, 2003). Deficiency in spatial learning and memory is obvious in rats treated with MDMA (Sheng, et al., 1996; Gibb, Johnson, & Hanson, 1990; Steranka & Rhind, 1987).

In human abusers of MDMA, the most consistent finding is the impairments in short-term memory (Bolla, McCann, & Ricaurte, 1998; Vorhees, Reed, Skelton, & Williams, 2004; Able, Gudelsky, Vorhees, & Williams, 2006). It is shown that the multiple-time administration of MDMA produces persistent deficiency in biochemical markers of 5-HT axon terminals in some brain areas such as hippocampus (Green, et al., 2003; Able, et al., 2006).

Despite the persistent effects of MDMA on 5-HT axon terminals, there is evidence that MDMA produces neuronal degeneration within the hippocampus (Kermanian, et al., 2012; Warren, et al., 2007). The mechanism of MDMA-induced depletion of the central nervous system (CNS) serotonin (5-hydroxytryptamine, 5-HT) is believed to involve the generation of Reactive Oxygen Species (ROS) (Broening, et al., 1995; Kermanian, et al., 2012; Warren, et al., 2007). The process of apoptosis is controlled by a diverse range of cell signals, which may originate either extracellularly via extrinsic inducers, or intracellularly via intrinsic inducers. The involvement of ROS at different phases of the neuronal apoptotic pathways is clearly established (Maycotte, Guemez-Gamboa, & Moran, 2012).

Extracellular adenosine acts via receptors coupled with G-protein (adenosine receptor subtypes A1, A_2A_, A2B, and A3) and exerts diverse physiological effects (Fredholm, Jacobson, Klotz, & Linden, 2001). A number of cellular components regulate apoptosis. The Bcl-2 protein can inhibit apoptosis by direct action, while Bax and/or Bak promote apoptosis (Kuwana & Newmeyer, 2003). The current study already found that A_2A_ agonist and CGS treatment may protect against MDMA induced apoptosis in striatum (Soleimani, Katebi, Alizadeh, Mohammadzadeh, & Mehdizadeh, 2012). The current study aimed at investigating the interaction between A_2A_ receptor and MDMA treatment at the molecular level in hippocampus.

## 2. Methods

### 2.1. Drugs and chemicals

MDMA hydrochloride, and other reagents used in the present experiment were purchased from Sigma Chemical (Sigma, LaJola, CA, USA.); the adenosine A_2A_ receptor agonist, (2-[p-(2-carboxyethyl) phenethylamino]-5′-N-ethylcarboxamidoadenosine) (CGS 21680), and the A_2A_ receptor antagonist [7-(2-phenylethyl)-5-amino-2-(2-furyl-) pyrazolo-[4,3-e]-1,2,4 triazolo [1,5-] pyrimidine] (SCH58261) were purchased from Tocris Cookson (Ball-win, MO, USA). CGS and SCH in a dose of 0.03 mg/kg body weight dissolved in 10% dimethylsulfoxide (DMSO) were administered intraperitoneally (i.p.) in animals.

### 2.2. Animals

A total of 40 adult male Sprague-Dawley rats (Pasture Institute, Tehran, Iran), weighted 200 to 500 g were used in the current study. Rats were maintained at the animal house under standard conditions (food and water ad libitum, 12:12 hours light/dark cycle, 21±3°C). All experiments involving rats were approved by the Animal Care Committee of Iran University of Medical Sciences, Tehran, Iran. Rats were randomly assigned into 4 experimental groups (n=10): Control: 10% DMSO 1 mL/kg, i.p., once a day for seven days. Treatment I: MDMA 10 mg/kg, i.p., once a day for seven days. Treatment II: MDMA+CGS, i.p., once a day for seven days. Treatment III: MDMA+SCH, i.p., once a day for seven days.

At the end of the seventh day, 5 rats in each group were decapitated and perfused. Their brains were removed for terminal deoxynucleotidyl transferase-mediated dUTP nick end labeling (TUNEL) test. TUNEL is a widely used method to detect apoptotic cells in tissue sections. Five rats in each group were killed and their brains were removed for the Western blotting study.

### 2.3. Tissue preparation

#### 2.3.1. TUNEL staining

Rats were anesthetized with ketamine (100 mg/kg, i.p.) and xylazine (10 mg/kg, i.p.) and, then, an incision was made on the skin to expose heart. Another incision was made on left ventricle to enter the perfusion tube; 10 to 150 mL of normal saline with 0.1 mL of heparin perfused to remove blood from vessels, followed by 150 to 200 mL of paraformaldehyde 4% in 0.1 M/L phosphate buffer (pH 7.4) as fixative solution. Then, whole brains were extracted. Tissue was processed for paraffin embedding and sagittally sectioned at 5 μm. The sections were deparaffinized and dehydrated by heating at 60°C in oven for 60 minutes and, then, rehydrated by xylol and graded ethanol solution, respectively.

Then, the histological samples were incubated in proteinase K (15 μg/mL) for half an hour. After that, the sections were quenched in 3% hydrogen peroxide/methanol for 10 minutes, in dark at room temperature. After 3 washes in Tris wash buffer (each 5 minutes), the sections were incubated with TUNEL reaction mixture for 1 hour at 37°C. Sections were washed in Tris wash buffer 3 times for 5 minutes each and, then, incubated with POD for 15 minutes at 37°C. Again, sections were washed in Tris wash buffer 3 times for 5 minutes each and, then, color development was performed in the dark room with DAB for 15 minutes. Then, hematoxylin solution was used as counter stain. After washing in Tris wash buffer 3 times for 5 minutes, the number of TUNEL positive CA1 neurons per mm length of the medial CA1 pyramidal cell layer was counted carefully in 5 sections per animal. Cell counts from the hippocampus on each of the 5 sections were averaged to provide the mean value.

#### 2.3.3. Western blot analysis

After anesthesia, craniotomy was performed and the brain was removed and placed on ice. The meninges were removed and hippocampus was separated from the hemispheres, snapped frozen in liquid nitrogen, and stored at −70°C. Collected tissues were homogenized in an ice-cold homogenizing buffer (50 mM Tris-HCl, 150 mM NaCl, 1 mM ethylenediaminetetraacetic acid (EDTA), and 0.5 mM Triton X-100, pH 7.4) and protease inhibitor cocktail tablets (Roche, Germany) for 1 hour and, then, were centrifuged (Eppendorf, Hamburg, Germany) at 12 000 g for 20 minutes at 4°C. The supernatant was removed and the protein concentration was determined with a Bio-Rad assay system (Bio-Rad, San Francisco, CA, USA). The protein extracts (10 μg) were run on a 10% sodium dodecyl sulfate polyacrylamide gel electrophoresis (SDS–PAGE), and electroblotted on to nitrocellulose membranes (Millipore, USA).

The membranes were, then, stained with washable Ponceau S solution to confirm equal protein loading. After washing the membranes with distilled water, they were blocked with Tris-buffered saline containing 0.02% Tween-20 and 5% of nonfat milk. Antibodies for Bax (mouse monoclonal, 1:1000 dilution; Beyotime Biotech) and Bcl-2 (mouse monoclonal, 1:1000 dilution; Beyotime Biotech) were applied at 4°C. The blots were, then, washed and incubated with respective alkaline phosphatase-coupled secondary antibodies (Bio-Rad) at 1:10000 dilutions. After extensive washing, the protein bands detected by the antibodies were analyzed. Values were compared using densitometric measurements using an image analysis system (UVI doc, Houston, Texas, USA) and explain by optical density (OD) ratio.

### 2.4. Statistical analysis

Data were shown as mean±structural equation modeling (SEM). All statistical analyses were conducted by SPSS software version 15. Differences between the groups were performed using one-way ANOVA and the Tukey test. A value of P<0.05 was considered statistically significant.

## 3. Results

### 3.1. CGS decreased and SCH increased the cell apoptosis induced by MDMA

**Figure 1:**
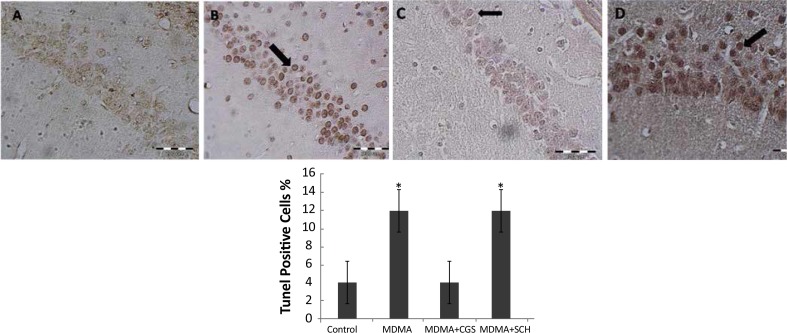
Tunel assay of hippocampi. A: control, B: MDMA, C: MDMA+CGS, D: MDMA+SCH groups. Black arrow shows apoptotic cells. *: P>0.05

**Figure 2:**
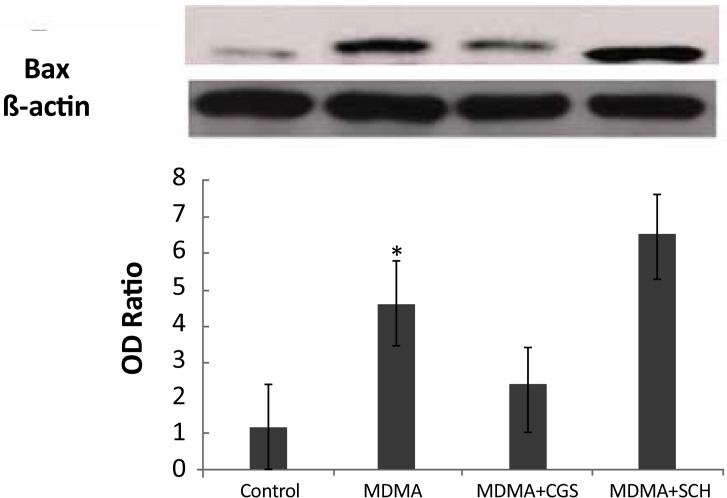
Western blot analysis of BAX expression in the rat hippocampi. *: P>0.05

The number of TUNEL-positive cells in MDMA treated rats were significantly higher than those of the control group (P<0.05). Interestingly, the number of TUNEL-positive cells significantly reduced in CGS group compared to MDMA group (Figure 1). The number of TUNEL-positive cells in rats with antagonist treatment (SCH) significantly increased when compared to that of MDMA group (P<0.05) (Figure 2).

### 3.2. Involvement of Bcl-2 and Bax Proteins in the cell apoptosis-induced MDMA

**Figure 3:**
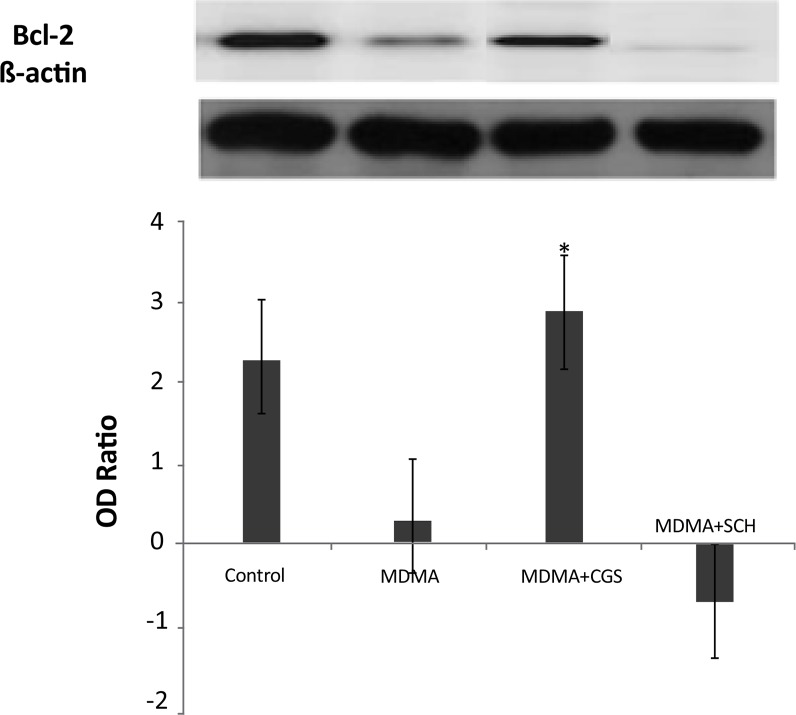
Western blot analysis of BCL-2 expression in the rat hippocampi. *: P>0.05

The expression of bcl-2 significantly increased in MDMA+CGS group, compared with MDMA group (P<0.05). The expression of bcl-2 significantly decreased and bax expression significantly increased in MDMA group, compared with the control group (P<0.05). The bax expression increased in rats with antagonist treatment (SCH), compared with MDMA group (Figure 3).

The data are shown as means±SEM. The number of rats in each group was 5. The data were analyzed by oneway ANOVA, followed by the Tukey Test (P<0.05).

## 4. Discussion

The current study investigated the influence of adenosine receptor agonist and antagonist on the MDMA induced apoptosis in hippocampus. A major finding of is the current study was that MDMA can cause apoptosis intra hippocampus mediated by A_2A_ adenosine receptors via mitochondrial pathways. Schmued and Bowyer (1997) showed that in 70% of the mice, degeneration of neurons occurred in the different species within 5 days after methamphetamine administration, which occasionally took place in the hippocampus and cortex (Schmued & Bowyer, 1997). In fact, it was shown that methamphetamine (METH)-induced increases in TUNEL-positive cells, and caspase-3 activation, and poly (ADP-ribose) polymerase (PARP) cleavage are all attenuated in Cu/Zn-SOD mice (Deng & Cadet, 2000).

The current study found similar results showing that the number of TUNEL-positive cells in MDMA-treated rats significantly increased. It is known that bcl-2 family plays a key role in the apoptosis induced by amphetamines (Montiel-Duarte, et al., 2002; Capela, et al., 2007). MDMA induces modifications in the expression of the splice variants of the bcl-x gene in neurons (Stumm, et al., 1999). The fact was that MDMA diminished Bcl-XL protein levels pointed out to mitochondria as a target for its pro-apoptotic effect, since Bcl-2 family proteins modulate the permeabilization of mitochondrial membranes and the subsequent liberation of pro-apoptotic factors such as cytochrome C (Tsujimoto, Shimizu, Narita, & Tsujimoto, 1999). Bcl-2 inhibits the release of the cytochrome C from mitochondria and cause inhibition of caspase activation and apoptosis (He, Xu, Yang, Zhang, & Li, 2004).

It was previously reported that METH induces significant increases in the pro-death bcl-2 family genes bad, bax and bid, and decreases in the anti-death genes bcl-2 and bcl-xl (Cadet, Jayanthi, & Deng, 2005; Jayanthi, Deng, Bordelon, Mccoy, & Cadet, 2001). Moreover, an increase caspase-3 activity was reported in the hippocampus of rats followed by a neurotoxic does of MDMA (Tamburini, 2006). The current study results suggested that MDMA administration might cause increases in the pro-death/anti-death ratio of the bcl-2 family of genes leading to apoptosis in hippocampus.

In agreement to the current study results, it was already reported that METH treatment downregulated bcl-2 in the striatum in mice (Imam, et al., 2001). Authors’ previous study described that coadministration of MDMA with A_2A_ adenosine antagonist, SCH produces a proapoptotic effect, increasing the expression of bax mRNA in rat striatum (Cadet, Ordonez, & Ordonez, 1997). The current study found that the number of TUNEL-positive cells in CGS and MDMA treated group significantly reduced, compared to that of MDMA-treated group. Decrement of apoptosis after the use of A_2A_ receptor agonists are related to the changes of bax and bcl-2 expression that protect neurons after ischemia (Rosin, Hettinger, Lee, & Linden, 2003; Zamani, et al., 2013; Soleimani, et al., 2012; Zamani, Katebi, Mehdizadeh, Mohamadzadeh, & Soleimani, 2012) It was already reported that increased expression of bcl-2 can prevent apoptosis of immortalized neuron cells by methamphetamine (Rosin, et al., 2003).

Jayanthi et al. (2001) reported that injection of methamphetamine causes the activation of apoptotic pathways such as upregulation of bax and downregulation of bcl-2. It was in agreement with the current study results describing that MDMA treatment cause upregulation of bax and downregulation of bcl-2. The presented results significantly reversed by adenosine receptor agonists and intensified by adenosine receptor antagonists. Kermanian et al. (2012) also indicated that A_2A_ agonist can protect against MDMA neurotoxic effects. The current study results suggested that MDMA induced apoptosis intra hippocampus was partly mediated via the mitochondrial pathway and was significantly associated with the adenosine A_2A_ receptors. These data might suggest that adenosine agonist could be used as an agent to treat MDMA induced neurotoxic effects.

The current study investigated the involvement of the selective adenosine A_2A_ receptor agonists in the development of apoptosis associated with acute MDMA injection. The results indicated that stimulation of the adenosine A_2A_ receptor plays a certain role in modulating the neuroadaptive changes appearing during MDMA treatment, and that the adenosine A_2A_ receptor agonists may serve as useful drugs to protect MDMA injury. Hence, the current investigation introduced adenosine A_2A_ agonists as possible vehicles for pharmacotherapy of MDMA dependence.
